# Garlic Accelerates Red Blood Cell Turnover and Splenic Erythropoietic Gene Expression in Mice: Evidence for Erythropoietin-Independent Erythropoiesis

**DOI:** 10.1371/journal.pone.0015358

**Published:** 2010-12-29

**Authors:** Bünyamin Akgül, Kai-Wei Lin, Hui-Mei Ou Yang, Yen-Hui Chen, Tzu-Huan Lu, Chien-Hsiun Chen, Tateki Kikuchi, Yuan-Tsong Chen, Chen-Pei D. Tu

**Affiliations:** 1 Institute of Biomedical Sciences, Academia Sinica, Taipei, Taiwan Authority; 2 Department of Molecular Biology and Genetics, Izmir Institute of Technology, Urla, Turkey; 3 National Genotyping Center, Academia Sinica, Taipei, Taiwan Authority; 4 Taiwan Mouse Clinic, National Phenotyping Center, Academia Sinica, Taipei, Taiwan Authority; 5 Department of Biochemistry and Molecular Biology, The Pennsylvania State University, University Park, Pennsylvania, United States of America; University of Louisville, United States of America

## Abstract

Garlic (*Allium sativum*) has been valued in many cultures both for its health effects and as a culinary flavor enhancer. Garlic's chemical complexity is widely thought to be the source of its many health benefits, which include, but are not limited to, anti-platelet, procirculatory, anti-inflammatory, anti-apoptotic, neuro-protective, and anti-cancer effects. While a growing body of scientific evidence strongly upholds the herb's broad and potent capacity to influence health, the common mechanisms underlying these diverse effects remain disjointed and relatively poorly understood. We adopted a phenotype-driven approach to investigate the effects of garlic in a mouse model. We examined RBC indices and morphologies, spleen histochemistry, RBC half-lives and gene expression profiles, followed up by qPCR and immunoblot validation. The RBCs of garlic-fed mice register shorter half-lives than the control. But they have normal blood chemistry and RBC indices. Their spleens manifest increased heme oxygenase 1, higher levels of iron and bilirubin, and presumably higher CO, a pleiotropic gasotransmitter. Heat shock genes and those critical for erythropoiesis are elevated in spleens but not in bone marrow. The garlic-fed mice have lower plasma erythropoietin than the controls, however. Chronic exposure to CO of mice on garlic-free diet was sufficient to cause increased RBC indices but again with a lower plasma erythropoietin level than air-treated controls. Furthermore, dietary garlic supplementation and CO treatment showed additive effects on reducing plasma erythropoietin levels in mice. Thus, garlic consumption not only causes increased energy demand from the faster RBC turnover but also increases the production of CO, which in turn stimulates splenic erythropoiesis by an erythropoietin-independent mechanism, thus completing the sequence of feedback regulation for RBC metabolism. Being a pleiotropic gasotransmitter, CO may be a second messenger for garlic's other physiological effects.

## Introduction

For millennia, garlic (Allium sativum) has been valued in many cultures both for its health effects and as a popular culinary flavor enhancer. Garlic's chemical complexity is widely thought to be the source of its many health benefits, which include, but are not limited to, anti-platelet, pro-circulatory, anti-inflammatory, anti-apoptotic, neuro-protective, and anti-cancer effects [1]–[3]. Although the garlic formulation of AGE (aged garlic extract) has a significant anti-oxidant activity on sickle RBC, excessive garlic intake in animals often leads to hemolytic anemia [4]. While a growing body of scientific evidence strongly upholds the herb's broad and potent capacity to influence health, the common mechanisms underlying these diverse effects remain disjointed and relatively poorly understood. Specifically, the possible relationship between erythropoiesis and garlic's health benefits has not been investigated. In light of the uncertainty regarding garlic's active ingredients [1] and the formation of H_2_S from garlic's organosulfides in isolated aorta sections [5], we wish to address the possibility of a second messenger for garlic's diverse physiological effects.

We have adopted a phenotype-driven approach to investigate the effects of dietary garlic on erythropoiesis in a mouse model. The array of phenotypic findings—histochemical analyses of spleens, examination of RBC morphologies, measurements of RBC half-lives, immunobloting, and mRNA expression profile analysis—all affirm that garlic accelerates RBC turnover and enhances splenic erythropoiesis and related gene expression. Our findings point toward a robust model for illuminating garlic's diverse health benefits. We propose that increased CO production by the heme oxygenase pathway stimulates splenic erythropoiesis by an erythropoietin-independent mechanism as a general homeostatic response to the accelerated RBC turnover caused by garlic consumption. Our proposal is supported by the fact that chronic exposure of mice on a garlic-free diet to low doses of CO stimulates erythropoiesis despite a decreased plasma erythropoietin level. We also propose that CO, a pleiotropic gasotransmitter [6]–[9], may also contribute to other physiological effects of garlic, in addition to erythropoietin-independent erythropoiesis.

## Materials and Methods

### Ethics statement

Animal use was approved by the IACUC of Academia Sinica and guided by international standard regulations for animal welfare (#RMiIBMTC2008014 on October 7, 2008).

### Diet components and reagents

Garlic and soybean oil were purchased from Taipei's supermarkets. Dietary ingredients were obtained from suppliers in France, UK, and USA. Antibodies were purchased from Abcam (Hsp70), Abnova (Klf1), Aviva Systems Biology (Tal1), Calbiochem (HO-2), Chemicon International (HO-1; Hif1α), Genscript (Gata-1), and Sigma-Aldrich (Sox6; GAPDH). Mice (6–8 week old male C57BL/6JNarl or B6) were purchased from the National Laboratory Animal Center in Nankang, Taipei, Taiwan.

### Diet preparation, mouse care, and experimental protocol

Mice were fed (*ad libitum*) a diet containing 200 g/kg (20%) vitamin free casein, 100 g/kg (10%) sucrose, 539.5 g/kg (53.95%) cornstarch, 70 g/kg (7%) soybean oil, 35 g/kg (3.5%) AIN93 regular mineral mix, 10 g/kg (1%) AIN93G vitamin mix, 2.5 g/kg (0.25%) choline bitartrate (41.1% choline), 3 g/kg (0.3%) L-cysteine, and the addition of either 40 g/kg (4%) cellulose (control or cellulose-supplemented diet) or 40 g/kg (4%) lyophilized husk-free raw garlic powder (garlic- supplemented diet) [10]. Male B6 mice (6–8 week old) were divided into two diet groups and maintained in the SPF (specific pathogen-free, barrier) facility in IBMS. Diet cups in each cage were replaced at least twice a week. Nine mice from each of the cellulose- or garlic-supplemented diet groups were randomly selected for analysis at time points of 0, 3, 6, 10, 15 weeks. Six of the nine mice were processed for histopathological examinations. The remaining three were sacrificed for tissues/organs, which were snap-frozen in liquid nitrogen for storage until use. The frozen tissues were used for RNA isolation (microarrays and qPCR), and immunoblots. CO treatment was carried out three times a week (*e.g*. MWF) in a clear Plexiglas box (inside dimension: 37×32×18 cm) under ∼250 ppm of CO (measured by a Bühler brand BA5000 CO analyzer) in the presence of a water bottle in the afternoons. Control treatment was carried out similarly with air delivered from a gas cylinder.

### Red blood cell morphology

RBC indices were determined with an Abbott Cell-Dyn 3700 analyzer. Blood (tail) collected in EDTA tubes was stained with 1% Brilliant cresyl blue [11]–[12] to count the percentage of biconcave and round-shaped, darkly-stained cells (1,000 cells per slide, 4 mice per diet group) at each time point. For accurate determination or differential counts of the variety of RBCs, blood was smeared on a glass slide and occasionally stained with the Giemsa method (Sigma GS500). Reticulocytes were photographed (5 regions per slide) after the blood smears were stained with new methylene blue under an Olympus BX51 microscope at 100×. The number of reticulocytes were counted and calculated among ∼1,000 RBCs.

### Scanning electron microscopy (SEM) of red blood cells

RBCs were washed with 0.1 M sodium cacodylate buffer (pH 7.4) three times, and then fixed with 2% glutaraldehyde for 2 hours (room temp.). Cells were centrifuged, washed, dehydrated stepwise in graded alcohols, and then diluted 10-fold in dry acetone. A drop of the suspension was placed on a piece of cover glass. The cover glass was coated with gold in a sputter coater and examined in a JSM-T330A scanning electron microscope (JEOL Ltd. Tokyo, Japan) at 15 kV. Randomly selected fields of well spread cells were photographed at 5,000×.

### Histopathological Analyses

Perfusion of animals and preparation of paraffin sections were according to those in Kikuchi *et al* [11]. Paraffin sections of tissues/organs were stained with hematoxylin and eosin (H&E) for histopathological examinations [12]. Spleen sections were stained for iron with K_3_[Fe(CN)_6_] [13]. After identifying the red pulp region by Nomarsky prism attached to an Olympus light microscope, iron droplets (Prussian blue, KFe[Fe(CN)_6_]) were quantified with assistance of the MetaMorph Offline software (Molecular Devices). A pre-determined color threshold program was used for a set of slides in a given figure. The relative iron content was obtained from the ratio of integrated areas of Prussian blue spots to that of the selected red pulp region in the same slide. Spleen macrophages were visualized through the acid phosphatase activity with the Gomori's method [14].

### RBC half-lives

*N*-hydroxysuccinimide-biotin was injected into each mouse (n = 9 for each diet group) at week 12 of feeding [15]. Mice were bled at 36 hours after injection, then at 3-day intervals for the first two weeks, 3–4 day intervals for the third and fourth week, and weekly for 1–2 additional time points. We stained washed cells with streptavidin-phycoerythrin R-PE and analyzed the washed labeled cells on a FACSCalibur. The data set was analyzed by the NLREG algorithm, with approximation by Gauss-Newton and Levenberg-Marquardt methods [16]–[17]. The NLREG algorithm performs linear and non-linear regression analyses, surface and curve fitting. We used the NLREG algorithm to solve the equation, A(t) = A_0_ (1−t/T)e^−kt^ (T is the time of senescent death of mouse RBCs) [18]. The half lives are estimated when A(t) = A_0_/2 for each mouse. We then used the Wilcoxon two-sample rank-sum test (a nonparametric test) to evaluate the difference of the estimated half-lives of control and garlic-fed mice [19].

### RNA isolation, microarray and real-time PCR analyses

The RIN numbers of the samples were from 8 to 9.5 out of a scale of 10 (Bioanalyzer 2100). High-density microarray analyses of gene expression were carried out with week 15 RNA samples (n = 3 for each diet groups) [20] by the Affymetrix Gene Expression Service Lab of Academia Sinica. Microarray data were analyzed with the GeneSpring™ software (Agilent). One-way ANOVA analysis was used to determine statistically important differences in gene expression (*p*<0.05). Gene ontology analysis was performed to assign molecular functions. We used TaqMan® qPCR to validate selected candidate genes. cDNA was synthesized from the total RNA using a high-capacity cDNA Achieve kit (ABI). After removing contaminating genomic DNA with Turbo DNA-free™ kit (Ambion), qPCR was performed in duplicates on the three biological replicates (ABI 7900HT qPCR machine). All microarray data is MIAME compliant and that the raw data has been deposited in GEO (Accession number GSE10344).

### Preparation of total cell extracts and immunoblotting

We used the Complete Lysis Buffer (Active Motif) to prepare total cell extracts from frozen tissues (100 mg liver or 50 mg spleen). Proteins were determined by the BCA assay (Pierce) before immunoblotting (30 µg splenic proteins per lane on 0.75 mm thick SDS-PAGE, 29∶1, followed by transfer with a semi-dry procedure). After reactions with antibodies, color was developed with Fuji Image Systems LAS-3000 and signals quantitated by the MetaMorph Offline software. GAPDH (glyceraldehyde 3-phosphate dehydrogenase) served as the internal reference for each gel/membrane. Each comparison (garlic *vs* control) was based on the ratio of signals for a particular protein over GAPDH on the same filter. The fold of increase in garlic-fed samples was calculated as [(proteinX/GAPDH) in garlic-fed mouse spleen/(proteinX/GAPDH) in control mouse spleen].

## Results

**Figure 1:**
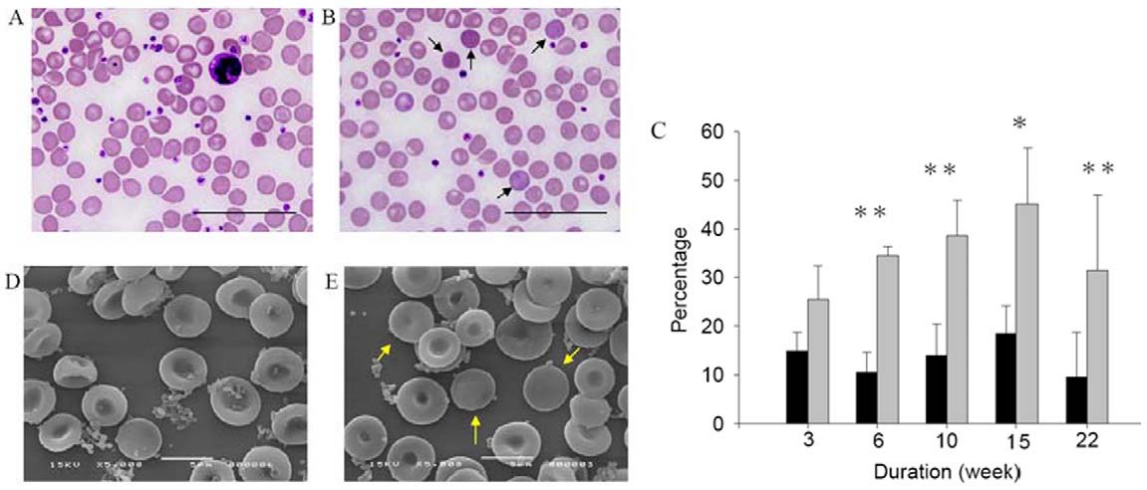
Morphology of RBCs from cellulose- (A, D) and garlic-supplemented (B, E) diet groups. RBCs were stained with Giemsa (week 6) to show darkly-stained cells (arrows in Panel B). (A) Most RBCs of control samples stained intensely around the edges rather than in the middle, reflecting their bi-concave shape. Among the uniform-sized RBCs are many small platelets and a neutrophil with a multi-lobed nucleus. (B) Darkly-stained RBCs in garlic-fed samples are non-nucleated, round or ovoid-shaped cells (arrows in Panel B) with almost the same size as the RBCs in control samples. They have more intense colors with a dark blue tint. (C) The % difference of darkly-stained cells at each time point (mean±s.e.m., n = 3–4, Brilliant Cresyl blue staining) between the two diet groups (control:black bars; garlic:gray bars) is significant (student's *t* test: *, *p*<0.05; **, *p*<0.01) throughout the time course. (D and E) SEM of RBCs (5000×) from control (D) and garlic-fed (E) mice (week 17). RBCs of garlic-fed samples contain more round-shaped cells (arrows in E) and those with a smaller dent at center. Scale bars: 100 µm (Panels A and B) or 5 µm (Panels D and E).

**Figure 2:**
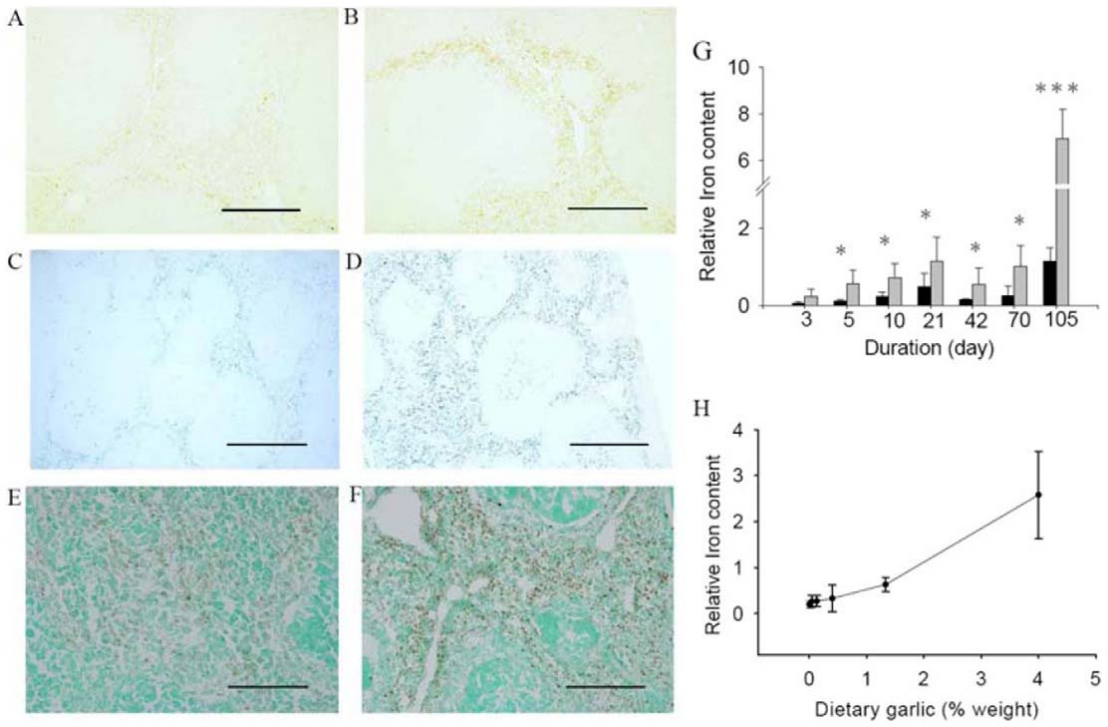
Histochemical phenotypes of spleens of C57BL/6J (n = 6) mice on cellulose-(A, C, E) and garlic-supplemented (B, D, F) diets (week 15). Spleen sections were shown with no staining (A, B) to reveal bilirubin, with Prussian blue stain for Fe^2+^(C, D), and with Gomori stain for macrophages *via* acid phosphatase and counter-stained for nuclei with methyl green (E, F). (G) Time course (n = 4) of Fe^2+^ accumulation in the spleens due to dietary garlic (4%). (H) Dose-response (n = 6) of dietary garlic in spleen sections. Scale bars represent 200 µm (A–F). (Statistics: *, *p*<0.05; ***, *p*<0.005, student's *t* test). Black bars: control; gray bars: garlic diet.

Our investigation on garlic's mechanisms of action began with blood analysis of tissue functions and histopathology examination of tissue/organ sections. The RBC indices and clinical chemistry profiles in mice from the two diet-groups (RBC counts, HGB, MCH, MCHC, MCV, PLT, Glc, total bilirubin (TBIL), direct bilirubin (DBIL), TCHO, TG) and marker enzymes (CRP, GGT, GOT, GPT, LDH) for organ functions—were nearly identical (Data not shown). The reticulocyte counts by new methylene blue stain for the two groups of mice were also similar at week 4 of feeding [5.4±1.5% s.d. for garlic-fed mice (n = 8) *vs*. 5.8±1.3% s.d. for cellulose control (n = 7)]. But we observed a higher proportion of darkly-stained blood cells—by Giemsa and brilliant cresyl blue staining [12]—in the blood of garlic-fed mice than in controls over a 22-week time course. These darkly-stained cells did not stain for DNA; they are most likely immature RBCs with more residual RNAs (Fig. 1B, dark blue tint). Examining the RBC preparations by scanning electron microscopy revealed that samples from garlic-fed mice contained more rounded RBCs among the typical bi-concaved ones (Fig. 1E). Their normal size indicated that these round RBCs are not the spherocytes often associated with anemia [21]. The garlic-fed mice were not anemic, nor did analyses reveal any increase in plasma erythropoietin (See below) and splenic Hif1α protein (data not shown), which indicates hypoxia. Surprisingly, we detected increased bilirubin (Fig. 2 A, B) and iron (Fig. 2C, D) in spleen sections of garlic-fed mice relative to the controls. Both iron and bilirubin (*via* biliverdin) are produced by the enzymatic degradation of heme, which accumulates in the spleen macrophages when aged and damaged RBCs are degraded [22]. The Prussian blue staining (Fig. 2G, H) for iron showed a color difference as early as 5–10 days in the spleens of garlic-fed mice in a dose-dependent manner (0.04% to 4% of garlic, w/w).

We suspected an accelerated RBC turnover in garlic-fed mice. This notion was affirmed by histochemical staining of spleen sections for acid phosphatase, a marker for macrophages [14]. The dark brown stains of the garlic-fed spleen sections were more pronounced than those of controls, indicating increased macrophages in the red pulp regions (Fig. 2E, F). This result supports our proposition on the increased RBC turnover in the spleens of garlic-fed mice.

To further substantiate this hypothesis, we compared the half-lives of RBCs between mice of the two diet groups. Analysis (week 12) of biotin-labeled RBCs in garlic-fed mice revealed that these cells have shorter half-lives relative to the cellulose-supplemented controls. The average half-life of RBC for the control mice is 17.9±3.3 days, compared to 13.2±1.9 days for the garlic-fed mice. We used the Wilcoxon two-sample rank-sum test (a nonparametric test) to evaluate the difference between the estimated half-lives of control and garlic-fed mice (n = 9 for each group) [16]–[19]. Given the null hypothesis that the RBC half-lives of control mice and the garlic-fed mice are equal, the two-tailed exact *p*-value equals 0.0054. [If we omit one outlier from each group due to incomplete injection of *N*-hydroxysuccinimide-biotin, the difference becomes 18.3±0.9 *vs*. 11.3±0.3 days with a two-tailed exact *p*-value of 6.2×10^−4^ (n = 8)]. Thus, the significance of the observed difference between the two half-lives is supported as the alternative hypothesis. Therefore, the increased turnover of RBCs in garlic-fed mice (over control) is due to shortened half-lives. In chemical terms, RBC turnover would release substantial amounts of heme (∼6×10^8^ heme molecules per RBC).

**Figure 3:**
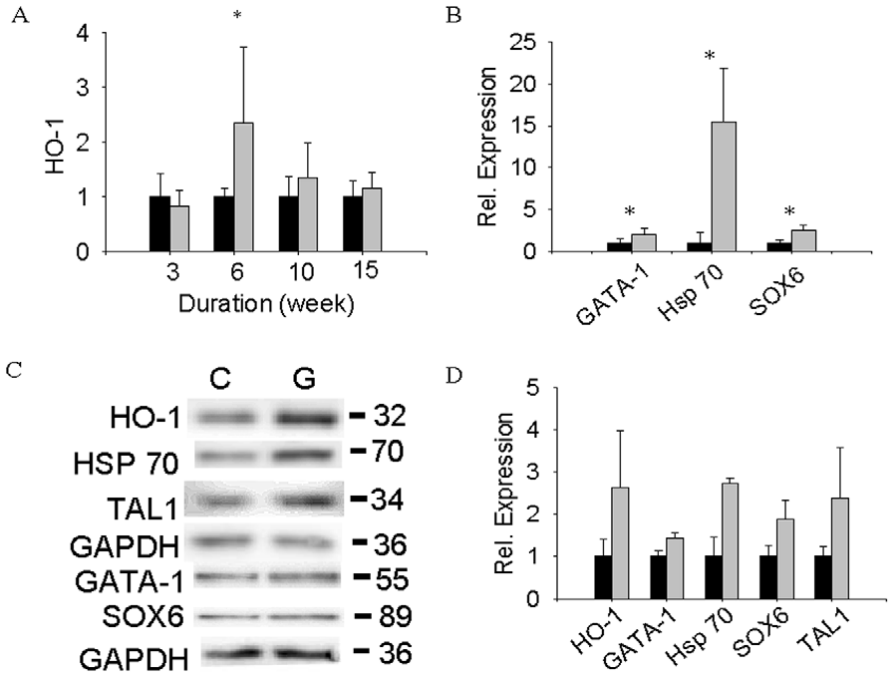
Splenic expression levels of HO-1, Hsp70, Gata-1, Sox6, and Tal1. (A) QPCR of HO-1 (mean±sd., n = 3). (B) qPCR of Gata-1, Hsp70, and Sox6 (mean±sd., n = 3). (C) Immunoblotting patterns of HO-1, Hsp70, Gata-1, Sox6 and Tal1 proteins in control (C) and garlic (G) spleen samples (week 15) with glyceraldehydes 3-phosphate dehydrogenase (GAPDH) as the reference protein. The numbers are in kilodaltons. (D) Quantification of results in C (mean±s.e.m., n = 3). Expression is calculated relative to the control samples of each time point (A) or of week 15 (B, D). The fold increase in garlic-fed samples was calculated as [ratio of (protein X/GAPDH) in garlic-fed mouse spleen]/[ratio of (protein X/GAPDH) in control mouse spleen]. (Statistics: *, *p*<0.05 by student's *t* test). Black bars, control diet; gray bars, garlic diet.

Heme released from RBC turnover should induce HO-1 heme oxygenase, which would then produce more Fe^2+^, biliverdin, and CO [23]–[24]. For this reason, we determined the HO-1 mRNA, HO-1 protein, and the constitutive HO-2 protein levels in the spleens of control and garlic-fed mice [25]. The maximal induction of HO-1 mRNA (2.3-fold, *p*<0.05) occurred around week 6 whereas the HO-1 protein level was elevated in garlic-fed spleens throughout the time course (∼2.5-fold at week 15, Fig. 3). In contrast, the constitutive HO-2 protein remained relatively constant (95±5% of control, n = 2). Thus, we conclude that increased degradation of heme occurred in garlic-fed mouse spleens, producing elevated levels of iron, bilirubin (Fig. 2 A–F), and presumably, a stoichiometric amount of CO [24]–[26].

How do garlic-fed mice avoid anemia with a shortened RBC half-life? We sought clues from the gene expression profiles in the spleens of the control and garlic-fed mice at week 15 (n = 3) (GEO accession number GSE10344). The patterns are consistent with the scheme of an increase in both RBC degradation and erythropoiesis in the spleens of garlic-fed mice. Among the up-regulated genes (garlic/control >1.8×, *p*<0.05) are myeloperoxidase (*Mpo*) and proteases (*Prss32, Prtn3*), which may participate in the turnover of RBC and hemoglobin to release heme. The released heme was then degraded by HO-1 to produce iron, bilirubin (*via* biliverdin), and CO (Figure 2). CO could then up-regulate Hsp70 through the MAPK pathway (p38β) and heat shock factor-1 (HSF-1) as demonstrated in murine lung endothelial cells and fibroblasts [27]. Hsp70 (15-fold increase of mRNA at week 15; *p*<0.05), which is stabilized by Hsp110 (5.5-fold elevation of mRNA at week 15, *p*<0.05; Table 1), can protect Gata-1, a key transcription factor in erythropoiesis, from proteolysis by caspase 3 [28]–[30]. Indeed, our results showed in garlic-fed mice an increase of Gata-1 mRNA peaking at week 6 (5-fold, *p*<0.05) and remaining high at week 15 (1.9-fold, *p*<0.05) (Table 1 and Fig. 3B). Additional evidence for transcriptional activation of erythropoiesis can be found in the increased levels of Tal1, which can form a complex with Gata-1 [29], and transcription factors Klf1 [31]–[32] and Sox6 [33], which were also elevated (Figure 3D).

**Table 1:** QPCR analyses of selected genes in mouse spleens (n = 3).

	Week 3		Week 6		Week 10		Week 15	
Gene Name	Cellulose	Garlic	Cellulose	Garlic	Cellulose	Garlic	Cellulose	Garlic
Hsp70	1.00±0.39	0.75±0.19	0.75±0.26	0.76±0.29	**0.36±0.09**	**0.61±0.23**	**0.24±0.07**	**3.69±3.20**
Hsp110	1.00±0.43	1.09±0.22	**1.12±0.69**	**2.56±1.00**	**0.69±0.28**	**1.73±0.78**	**0.54±0.13**	**2.96±1.90**
Klf1	1.00±0.69	0.91±1.15	**0.85±0.35**	**4.12±2.99**	**1.71±1.53**	**5.08±3.40**	**3.12±0.84**	**7.10±1.48**
Gata1	1.00±0.55	0.73±0.69	**0.61±0.18**	**3.02±2.27**	1.32±0.86	3.15±2.47	**2.29±1.09**	**4.43±1.80**
Sox6	1.00±1.01	2.07±1.37	**1.26±0.64**	**5.14±3.25**	**2.13±2.21**	**10.15±6.50**	**3.59±1.38**	**8.87±2.36**
Kel	1.00±1.17	2.18±1.48	**1.41±0.76**	**6.18±3.71**	2.72±2.71	10.12±8.72	**4.10±1.20**	**8.86±1.22**
Tfrc	1.00±0.40	1.09±0.35	**0.82±0.18**	**1.50±0.59**	**1.06±0.48**	**2.95±2.17**	**1.66±0.70**	**2.52±0.40**
Hmbs	1.00±0.65	1.78±1.24	**1.23±0.30**	**3.45±2.01**	**2.09±1.56**	**5.05±2.73**	**3.09±0.69**	**6.39±1.10**
Cpox	1.00±0.46	1.65±0.70	**1.07±0.28**	**3.05±1.57**	**1.39±0.64**	**3.23±1.94**	**2.27±0.88**	**4.08±0.53**
Alas	**1.00±0.22**	**0.68±0.19**	0.62±0.13	0.94±0.50	0.90±0.22	1.15±0.55	0.51±0.07	0.50±0.14
Uros	1.00±0.25	0.83±0.15	0.84±0.24	1.49±0.86	0.91±0.39	1.32±0.63	0.63±0.11	0.67±0.09
Hmox1	1.00±0.43	0.84±0.29	**0.66±0.11**	**1.55±0.92**	1.33±0.50	1.79±0.83	0.63±0.19	0.74±0.18
Check1	**1.00±0.17**	**1.54±0.26**	**1.04±0.20**	**1.57±0.42**	1.18±0.31	2.07±1.65	1.76±1.13	1.91±0.66
Ptdss2	**1.00±0.23**	**1.50±0.31**	**0.91±0.08**	**2.15±1.20**	1.69±0.48	2.90±1.57	**1.54±0.38**	**2.59±0.60**
Hey2	**1.00±0.22**	**0.67±0.19**	0.72±0.11	0.78±0.38	0.94±0.38	0.88±0.29	**0.45±0.11**	**0.29±0.05**

All data were normalized aganist Week 3 data (1.00) from control mice --- Numbers are mean±sd. **Bold numbers**: *P*<0.05 (student's t-test).

Evidence of increased splenic erythropoiesis in garlic-fed mice is further supported by the increase of mRNAs (>1.8-fold, *p*<0.05; microarrays) for cell cycle and related genes (cyclins A2, E1, E2, F…, aurora kinase A, centrosomal protein 76), and of mRNAs activated by Klf1 [32]–[33], such as those for heme biosynthesis (*Cpox and Hmbs*), structural components of RBC membrane (*e.g*. amino acid transporters, aquaporin 1, band 4.9, band 4.1, claudin 13, erythroid ankyrin 1, α_1_- and β_1_-spectrins, Kell blood group, Rhesus blood group, *etc*.), and those for the biosynthesis of glycerophospholipid (*e.g*. *Ptdss2*) (Table 1 and GSE10344). In contrast, Hey2 mRNA was down-regulated by ∼1.5-fold (*p*<0.05; Table 1). Hey2 mRNA is induced by hypoxia [34] so this down-regulation of Hey2 mRNA is again consistent with the absence of anemia in garlic-fed mice. Hey1, a paralog of Hey2, is known to interact with Gata-1 to inhibit erythropoiesis [35]. Overall, the increased expression of these genes in garlic-fed mice is consistent with the idea of stimulation of splenic erythropoiesis by garlic.

We also tested garlic's effect on the levels of Gata-1, Klf1, Sox6, Hsp110, Tfrc (transferrin receptor), and HO-1 mRNA expression to address possible changes of homeostatic erythropoiesis in the bone marrow. Whereas HO-1 showed no significant difference throughout, Gata-1, Klf1, Sox6, Tfrc, and Hey2 were ∼2- to 4-fold lower; Hsp110 continued to drop, to negligible levels at week 15 (n = 3, Table 2). Thus, dietary garlic enhanced erythropoiesis in the spleen but not in the bone marrow (Fig. 3, Tables 1 and 2).

**Table 2:** QPCR analyses of selected genes in mouse bone marrow (n = 3).

	Week 3		Week 6		Week 10		Week 15	
Gene Name	Cellulose	Garlic	Cellulose	Garlic	Cellulose	Garlic	Cellulose	Garlic
Gata1	1.00±0.85	0.86±0.71	**0.82±0.54**	**1.27±0.11**	0.66±0.18	0.80±0.28	**1.52±0.74**	**0.61±0.36**
Sox6	1.00±0.74	1.40±1.29	**0.96±0.45**	**1.76±0.34**	0.71±0.19	0.78±0.19	**1.65±0.53**	**0.83±0.29**
Tfrc	1.00±0.98	0.93±0.94	**0.58±0.33**	**0.95±0.20**	0.60±0.18	0.69±0.27	**2.40±1.22**	**1.25±1.07**
Hsp110	1.00±1.13	1.17±0.87	**0.32±0.15**	**0.18±0.01**	0.04±0.01	0.04±0.02	0.11±0.05	0.07±0.06
Klf1	1.00±0.85	0.76±0.65	**0.80±0.42**	**1.45±0.39**	0.68±0.17	0.66±0.17	**1.40±0.73**	**0.71±0.39**
Hmox1	1.00±0.01	0.71±0.66	0.89±0.76	1.45±0.69	0.33±0.09	0.36±0.09	1.05±0.54	0.76±0.70
Cpox1	1.00±0.96	0.99±0.97	**0.80±0.56**	**1.35±0.28**	0.68±0.23	1.09±0.60	2.25±1.24	1.52±1.65
Hey2	1.00±0.69	0.78±0.92	1.05±0.57	0.78±0.40	0.23±0.15	0.24±0.08	**0.88±0.94**	**0.21±0.09**

All data were normalized aganist Week 3 data (1.00) from control mice --- Numbers are mean±sd**. Bold numbers**: *P*<0.05 (student's t-test).

**Figure 4:**
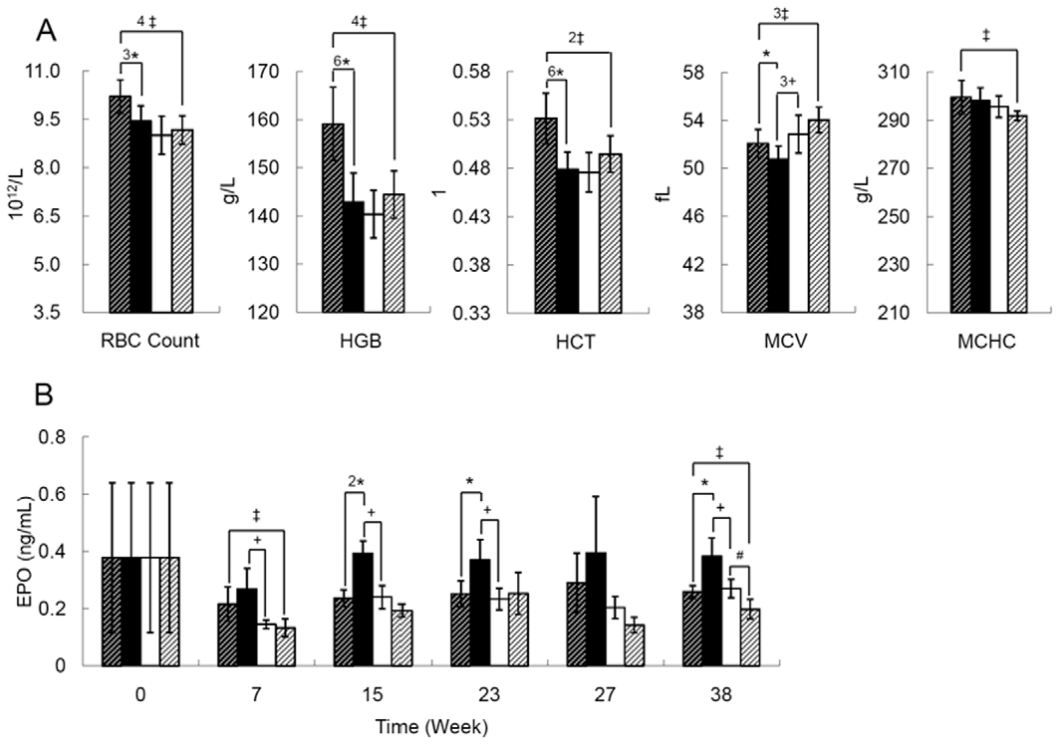
Evidence that CO treatment promotes increased erythropoiesis by an erythropoietin-independent mechanism. (A) Red blood cell indices of B6 mice on garlic-supplemented or garlic-free (cellulose-supplemented) diets with or without the treatment of CO at week 38: red blood cell concentration (RBC count, 10^12^/L), hemoglobin concentration (HGB, g/L), hematocrit (HCT, %), mean corpuscular volume (MCV, fL), mean corpuscular hemoglobin concentration (MCHC, g/L). Statistics: +, Air-Cellulose (AC) *vs*. Air-Garlic (AG); ‡, CO-Cellulose (COC)*vs*. CO-Garlic (COG); * CO-Cellulose (COC) *vs*. Air-Cellulose (AC); #, Air-Garlic (AG) *vs*. CO-Garlic (COG). One symbol, *p*<0.05; 2 symbols, *p*<0.01; 3 symbols, *p*<0.005; 4 symbols, *p*<0.001; 6 symbols, *p*<0.0001 (student’s *t* test); n = 7–10. The arrangements for each block of bars are (left to right): thick cross-hatched bars, cellulose diet and treated with CO (COC); filled bars, cellulose diet and treated with air (AC); open bars, garlic diet and treated with air (AG); thin cross-hatched bars, garlic diet and treated with CO (COG). (B) Plasma erythropoietin (EPO) levels (ng/mL), n = 4 for each set of samples at different point of the time course. Symbols are the same as those in Panel A. The pairwise comparisons between AC and COG are: week 7, *p*<0.013; week 15, *p*<0.003; week 23, *p* = 0.065; week 27, *p* = 0.084; week 38, *p*<0.007 (student's *t* test).

Since we cannot measure changes in CO concentration easily in garlic-fed mice, we instead inferred its production from the detection of increased Fe^2+^ and bilirubin [24]–[26]. To ensure that CO can indeed produce a systemic effect, we tested the effect of low dose CO (∼250 ppm in synthetic air: 21% O_2_ and 79% N_2_) on the RBC indices of mice. When mice breathed CO three times a week for 2 hours each session over 38 weeks, they manifested increased RBC counts (*p*<0.005, cellulose-supplemented diet), hemoglobin contents (*p*<0.0001), and hematocrit (*p*<0.0001). The average RBC volume (MCV = HCT/RBC count) was slightly increased (*p*<0.05) and the mean corpuscular hemoglobin concentration (MCHC = HGB/HCT) remained unchanged (Figure 4A, cellulose-supplemented diet). Garlic showed significant effect on MCV (*p*<0.005, garlic *vs* cellulose, no CO treatment) but suppressed CO's stimulatory effect, presumably through the increased RBC turnover (RBC count, *p*<0.001; HGB, *p*<0.001; HCT, *p*<0.01; CO treated mice, cellulose *vs* garlic diet).

Lastly, we determined the plasma erythropoietin (EPO) levels of different groups of mice in light of changes in RBC turnover rates. Both garlic-fed mice and CO-exposed mice showed lower plasma EPO levels than mice on cellulose-supplemented control diet. Furthermore, garlic-supplemented diet and CO treatment have additive effects to the reduction of plasma EPO levels (Figure 4B, mice on cellulose-supplemented diet treated with air (AC) *vs*. mice on garlic diet treated with CO (COG)).

## Discussion

Stimulation of erythropoiesis and related genes (Table 1) by garlic-fed mice is most likely mediated by CO, which by itself can increase the RBC counts, hemoglobin contents, and hematocrit in B6 mice (Figure 4A). It was also reported in rat that chronic exposure to CO increased erythropoiesis [36]. In our results (Figure 4B), however, plasma EPO levels of both garlic-fed mice and CO-treated mice on garlic-free (*i.e*. cellulose-supplemented) diet decreased relative to air-treated mice on cellulose-supplemented diet (control mice). Furthermore, the decrease of EPO showed an additive effect when garlic-fed mice were chronically exposed to CO (Figure 4B), supporting the proposal that garlic-fed mice produced extra CO. Nevertheless, none of these mice were anemic. Therefore, we have strong evidence (e.g. Figs. 3–4, Tables 1–2, GSE10344) for an EPO-independent pathway in the spleen, which might follow a combination of molecular events in three separate reports [27]–[28], [37].

Firstly, CO can stimulate HSF-1 activation through the MAPK pathway in the lung endothelial cells, resulting in the increased expression of Hsp70 and other heat shock proteins [27]. Our microarray expression profiles, qPCR, and/or immunoblots showed an increase of Hsp70 and Hsp110 (Figure 3 and Table 1) in the spleens of garlic-fed mice.

Secondly, increased Hsp70 can protect the key transcription factor for erythropoiesis, Gata-1, from caspase-3 mediated proteolysis [28]. Stabilization of Gata-1 presumably increased downstream gene transcription for erythropoiesis [30]. Indeed, we have observed an increase of Gata-1 and the downstream gene expression in our mRNA expression profiles and immunobloting results (Figure 3 and Table 1). Thirdly, the CO induced splenic erythropoiesis could be manifested through ERK1, a different subfamily member of the MAPK family, which is a negative regulator of the adult steady-state splenic erythropoiesis [37]. Ablation of ERK1 induces a splenic stress erythropoiesis phenotype, but the mice display no anemia and did not affect EPO levels or EPO/EPO receptor signaling [37]. Thus, CO could directly or indirectly inhibit ERK1 to induce EPO-independent splenic erythropoiesis. The major difference, however, is the lack of acute anemia in our garlic-fed animal model. The details of the proposed CO-mediated, EPO-independent erythropoiesis remain to be elucidated in garlic-fed mice.

The proposed increase of CO from faster RBC turnover by dietary garlic may also have other physiological functions, some of which may be important for garlic's many health benefits. It is widely perceived that garlic's various health effects stem from the synergistic actions among its diverse chemicals [1]–[3]. Our results on the mRNA expression profiles in the garlic-fed mouse spleens (GSE10344) indicate that the collective effects of phytochemicals (and their metabolites) on the expression of genes, for example, against oxidative stress, are very different from those reports using the alleged key derivatives of garlic such as diallyldisulfide, which changes the expression of cytochrome P450's and phase II enzymes at high doses [38]. We did not, to our surprise, observe much induction of mRNAs for detoxification enzymes by either increased iron or phytochemicals (or their metabolites) in the garlic-fed mouse spleens. Only the mRNAs of flavin containing monooxygenase 2, microsomal glutathione *S*-transferase (GST) 3, and GST Mu5 [39] were induced by dietary garlic (>1.8-fold, *p*<0.05). This suggests the absence of unusual oxidative stress in the garlic-fed mouse spleens. Such a condition could at least partially be attributable to the higher level of the potent anti-oxidant, bilirubin (and biliverdin), also produced from increased heme degradation [40]. Thus, chemoprevention mediated by induction of cytoprotective enzymes alone is not sufficient to account for the systemic health benefits of garlic [41].

A second mechanism by which garlic may exercise its health effect has been demonstrated in isolated aorta sections through the vaso-relaxation activities of H_2_S, which is produced from the organosulfur compounds of fresh garlic extracts, especially the allylsulfides [5], [42]. Their conversion to H_2_S is non-enzymatic and requires cellular GSH, L-cysteine, or protein thiols [42]. As an environment rich in reduced GSH, RBC would naturally convert garlic's organic polysulfides to H_2_S, which presumably can circulate to the whole body by binding to hemoglobin and other hemoproteins [5], [43]. Besides, the polysulfides may also modify surface and intracellular proteins by reacting with cellular protein thiols, leading to RBC turnover.

Our results point to a more robust biological amplification process that potentially provides a common second messenger for many of garlic's diverse physiological effects. Garlic and other plants of the genus *Allium* are known for their production of steroid saponins, compounds with hemolytic activities [1], [44]. Therefore, the presence of steroid saponins in garlic is consistent with the dose-response behavior in the Prussian blue stain of the spleen sections (Figure 2H); saponins could cause mild chronic hemolysis without anemia and resulting in accelerated RBC turnover. As the dosage of saponins (garlic) exceeds the rodent's erythropoietic capacity, anemia ensues [4]. It is entirely possible; however, other classes of garlic compounds manifested the phenotype in our study. We propose that accelerated RBC turnover is the crux of the mechanism(s) by which garlic sustains and amplifies its multiple biological effects. At garlic-enhanced rates of turnover, each degraded RBC may release up to ∼6×10^8^ heme molecules, of which a significant proportion are then converted to CO, to alleviate any heme toxicity [45]. Garlic's anti-platelet, procirculatory, anti-inflammatory, and anti-apoptotic effects parallel those reported for the HO-CO pathway for heme degradation [1], [6]–[9].

In the scheme of RBC turnover, CO can be regarded as a “feedback” regulator by stimulating splenic erythropoiesis, which differs from the EPO-dependent process in the bone marrow. In addition to its stimulatory effect on EPO-independent splenic erythropoiesis, CO is a pleiotropic gasotransmitter [6]–[9]. Furthermore, from the perspective of detoxification of garlic's phytochemicals, many of garlic's health benefits could be the manifestation of physiological recovery to achieve systemic homeostasis. This recovery is presumably mediated by a younger population of RBCs, induction of cytoprotective enzymes, H_2_S produced from garlic's polysulfides, and by CO (the heme oxygenase pathway), which significantly amplifies (up to ∼6×10^8^-fold) the chemical effects of garlic. The reticuloendothelial system of spleen apparently plays a crucial role in this recovery process. The relationship between the molecular details of CO-mediated pathways for erythropoiesis and of garlic's many health benefits remain to be elucidated.
